# Combined Use of Excimer Laser and High-Speed Rotational Atherectomy to Overcome a Severely Calcified Lesion in Endovascular Therapy

**DOI:** 10.1155/2019/1719035

**Published:** 2019-04-16

**Authors:** Keisuke Nakabayashi, Shinya Hata, Nobuhito Kaneko, Akihiro Matsui, Kazuhiko Tanaka, Hiroshi Ando, Minoru Shimizu

**Affiliations:** Kasukabe Chuo General Hospital, Heart Center, Saitama, Japan

## Abstract

Although endovascular therapy (EVT) is commonly used in treatment of peripheral artery disease (PAD), severely calcified lesions pose a challenge, in spite of the technical advancement. In this report, we discuss the case of a 74-year-old male with coronary artery disease and end-stage renal disease who presented at our institution with bilateral intermittent claudication. Angiography showed chronic total occlusion (CTO) of the right superficial femoral arteries (SFA). Because the bilateral external iliac arteries demonstrated moderate stenosis, we performed endovascular therapy on the right SFA-CTO using a contralateral approach. With the antegrade wire progressing into the subintimal space, direct distal-SFA puncture was performed and wire externalization was established. However, no devices (minimal balloon, microcatheter, or Crosser system) were able to pass the lesion in antegrade or retrograde manner, even though the child catheter support or needle cracking technique from outside/inside was applied. Therefore, we used a combination of an excimer laser and high-speed rotational atherectomy to overcome the severely calcified lesion. First, the excimer laser catheter (Turbo Elite 0.9 mm) ablated the entry to the CTO; however, it did not pass through completely. Thereafter, the thin microcatheter (Caravel) succeeded in crossing the CTO in an antegrade manner using the BAlloon Deployment using FORcible Manner (BADFORM) technique. After wire-exchange to the Rota-wire, rotational atherectomy (RotaLink Plus 1.5 mm) passed through the CTO. Subsequently, we could dilate the CTO lesion with a conventional balloon followed by bare metal stent deployment. The right ankle-brachial index of the patient improved from being unmeasurable to 0.79, and the intermittent claudication disappeared. This combination therapy, described as the “RASER” technique in coronary section, is accepted for reimbursement. However, these devices in EVT section are considered off-label use in Japan. Therefore, we have to refrain from frequent use of this strategy; however, this method provides an option for severely calcified lesions.

## 1. Introduction

Endovascular therapy (EVT) has developed over the past decade because of advancement in techniques and devices [1, 2]. Therefore, the clinical indication of EVT for peripheral artery disease (PAD) becomes widely spread. However, severely calcified lesions still remain challenging. To overcome this situation, some techniques and devices have been developed. Excimer laser atherectomy (ELA) is a unique procedure [3] that modifies the morphology of the surface-plaque as well as the hard tissues underneath the calcification to facilitate easy device crossing, balloon dilatation, and subsequent stenting. High-speed rotational atherectomy (RA) with Rotablador (Boston Scientific, Natick, MA, USA) is also an important device that modifies and debulks the calcified lesion in the coronary section [4]. These two options complete each other by utilizing their characteristics.

Here, we report the case in which combination therapy using ELA and RA successfully treated chronic total occlusion (CTO) of the superficial femoral artery (SFA) with severe calcification.

## 2. Case Presentation

**Figure 1:**
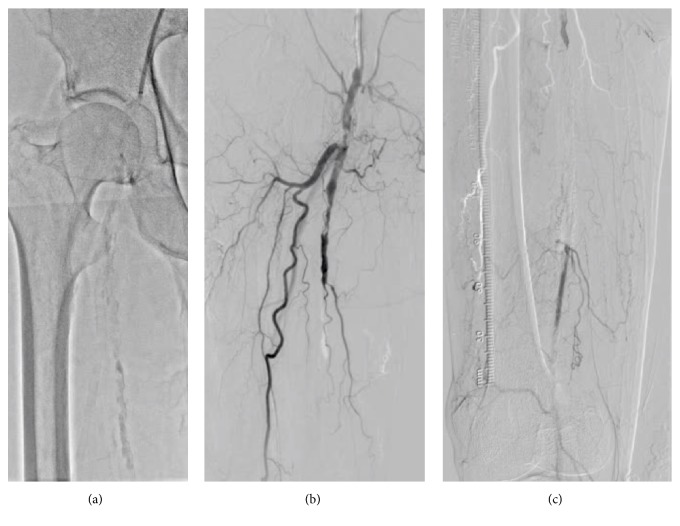
The first angiogram. (a) Digital angiography shows severe calcification of the right superficial femoral artery. (b) Digital subtraction angiography of the proximal part indicates the chronic total occlusion of the middle superficial femoral artery. (c) Digital subtraction angiography of the distal portion presents the puncturable distal superficial femoral artery.

**Figure 2:**
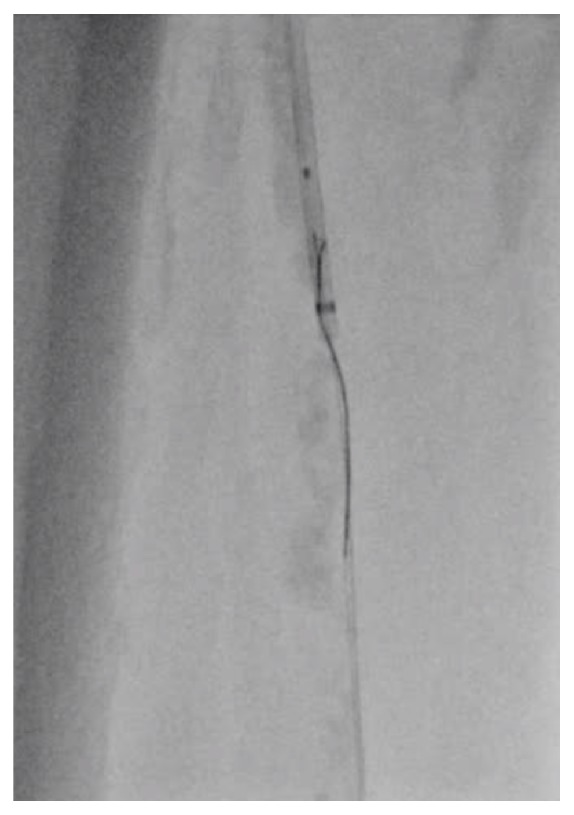
Rendezvous technique. The retrograde wire progresses into the antegrade child catheter.

**Figure 3:**
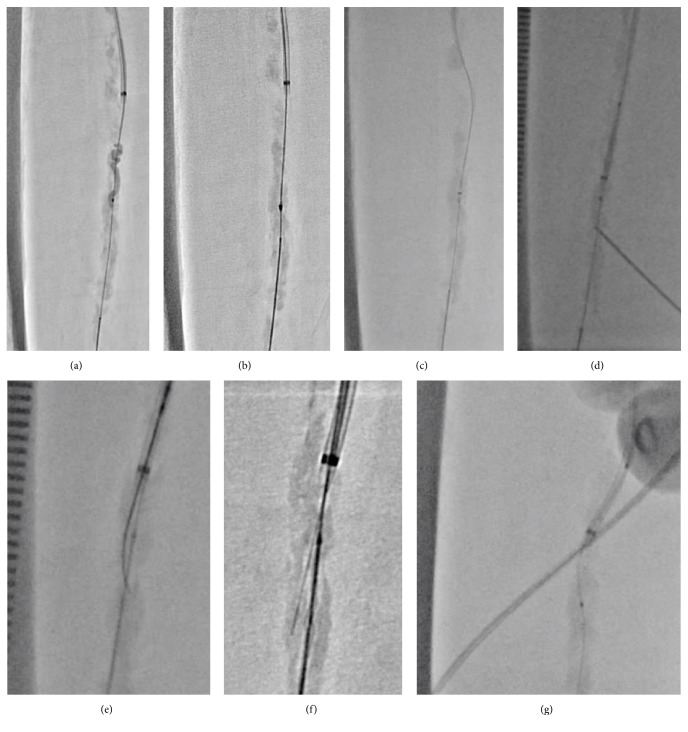
Devices that failed to cross the lesion. (a) The thin balloon is bent. (b) The Corsair PV microcatheter is fractured. (c) Crosser system cannot pass the lesion. (d) The needle cracking technique from outside of the vessel. (e) Needle cracking technique from inside of the vessel using the stiff wire. (f) Needle cracking technique from inside of the vessel using the tail of the wire. (g) The thin balloon cannot pass the lesion even after these devices and techniques.

A 74-year-old male with hypertension, dyslipidemia, diabetes mellitus, coronary artery disease, and end-stage renal disease was transferred to our institution due to the bilateral intermittent claudication 6 months before admission. He had been given 75 mg of clopidogrel and 200 mg of cilostazol per day. His right ankle-brachial index was unmeasurable, and echography was suggestive of right SFA-CTO. Although this lesion was classified as class D based on the Transatlantic Inter-Society Consensus Document (TASC) [5], the patient refused surgical revascularization. Therefore, we chose endovascular therapy (EVT) and obtained a written informed consent from the patient. Since echography also indicated moderate stenosis of the bilateral iliac arteries, we initiated EVT in the right SFA-CTO using the contralateral approach. The first angiogram revealed the severely calcified SFA-CTO (Figure 1; Video Clip S1). The antegrade approach with the stiff CTO wire resulted in the subintimal wiring. Therefore, we used the bidirectional approach with direct distal-SFA puncture. Finally, the retrograde-antegrade rendezvous technique led to wire externalization (Figure 2; Video Clip S2). Regardless of the strong back-up force by wire externalization and child catheter support, no devices were able to pass the lesion by the antegrade and retrograde approaches and the thin balloon catheter was bent, Corsair microcatheter (ASAHI Intecc, Aichi, Japan) was fractured, Crosser system (C.R.Bard, NJ, USA) could not pass the lesion, and needle cracking technique [6] from outside and inside of the vessel could not modify the lesion fully. Despite using all these techniques, a minimal balloon could not pass the lesion (Figure 3). We consider abandonment and elective surgical conversion; however, bleeding from the retrograde puncture point and needle cracking techniques was uncontrollable. Therefore, we had to cross the lesion to perform balloon-assisted hemostasis.

**Figure 4:**
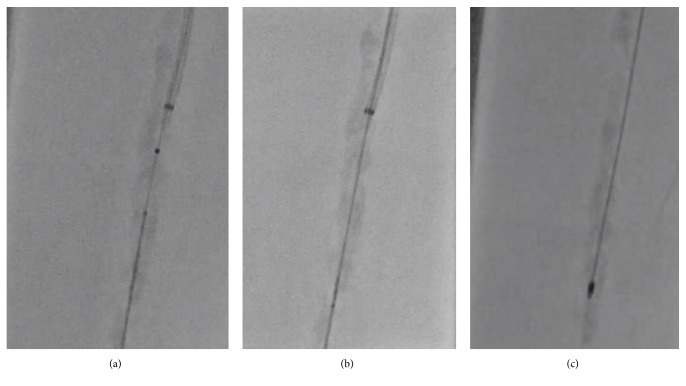
Combined strategy of the excimer laser atherectomy and high-speed rotational atherectomy. (a) The excimer laser catheter (Turbo Elite 0.9 mm) also cannot cross the lesion; however, it modifies the morphology of the chronic total occlusion lesion. (b) The Caravel microcatheter can pass the lesion. (c) Exchanging to the 0.009-inch Rota-wire from the conventional 0.014-inch wire, 1.5 mm burr ablation is performed.

Hence, we applied the ELA using the Turbo Elite 0.9 mm (Spectranetics, Co., USA). This device also could not pass the lesion; however, it modified the CTO entry-morphology (Figure 4(a)). After ELA ablation, the Caravel microcatheter (ASAHI Intecc) finally crossed the lesion using an antegrade approach with the BAlloon Deployment using FORcible Manner (BADFORM) technique [7] (Figure 4(b)), which enabled change of the wire from conventional the 0.014-inch wire to 0.009-inch RotaWire floppy (Boston Scientific) and RotaLink Plus 1.5mm burr ablation (Boston Scientific) (Figure 4(c)).

**Figure 5:**
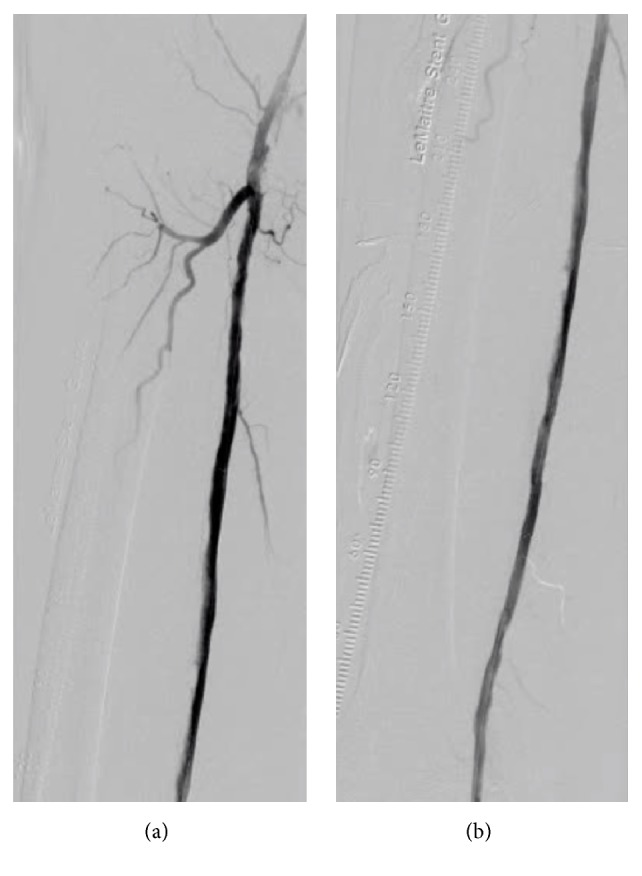
The final angiogram. (a) Digital subtraction angiography of the proximal superficial femoral artery. (b) Digital subtraction angiography of the distal superficial femoral artery.

Thereafter, we dilated the entire SFA lesion with conventional balloons and simultaneously achieved hemostasis of the distal-SFA puncture point. A bare nitinol stent was deployed due to severe dissection of the partial SFA. The final angiography revealed acceptable result without delay in blood-flow or bleeding complications (Figure 5; Video Clips S3 and S4).

Thereafter we treated the bilateral iliac arteries using the bare nitinol stents. The right ABI of the patient improved from being unmeasurable to 0.79, and his intermittent claudication disappeared. His ABI and symptom has remained satisfactory eight months after current EVT.

## 3. Discussion

The application of EVT is widespread with novel techniques and devices; however, severely calcified lesion is still a vulnerable point. To overcome this lesion, several atherectomy devices have been developed [8]. Most of these devices had relatively large diameters and posed problems with crossing the lesion. The combined strategy shown here could be superior in this point.

The application of ELA in EVT has been mainly limited to in-stent restenosis (ISR) lesions. The first prospective, multicenter study of 90 patients with ISR of nitinol stents in the femoropopliteal arteries treated by ELA and adjunctive percutaneous transluminal angioplasty (PTA) showed 97% procedural success, 7% residual stenosis, 2.2% 30-day major complication rate, and 64% 1-year freedom from target lesion revascularization [9]. A randomized control trial demonstrated the superiority of ELA with PTA versus PTA alone for treating femoropopliteal ISR in mid-term (6-month freedom from target lesion revascularization; 73.5% versus 51.8%, p < 0.005) [10]. With respect to imaging, intravascular ultrasound assessment indicated that ELA of peripheral artery lesions resulted in significant plaque debulking and increased lumen diameter with negligible degree of adventitial layer injury [11]. Angioscopy assessment also visualized the ELA effect of vaporization of thrombi in femoropopliteal in-stent lesions [12]. Thus, ELA was used as a debulking device. However, we herein used ELA as a modification or penetration device to cross the microcatheter to the distal CTO.

The application of high-speed RA in EVT section has been limited to a few case reports. Fukuda et al. reported a case of calcified SFA lesion after femoropopliteal bypass grafting treated by RA [13]. A guide wire and microcatheter crossed the lesion; however, a balloon catheter could not pass in their case. Hara et al. discussed the application of RA in heavily calcified CTO of the common iliac artery in a long-term hemodialysis patient. They had difficulties in passing the conventional microcatheter and used a Tornus microcatheter (ASAHI Intecc) instead [14]. The disadvantage of RA is the need for a dedicated 0.009-inch wire, which is cumbersome to control as a bare wire and requires microcatheter crossing.

**Figure 6:**
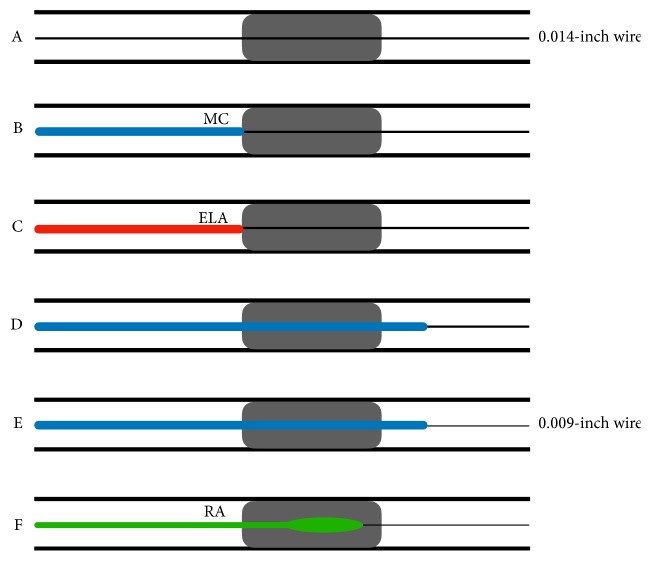
The schema of the “RASER” technique. A: conventional 0.014-inch wire crosses the severely calcified lesion. B: microcatheter often cannot pass the lesion. C: excimer laser catheter ablates and modifies the entry of the lesion. D: this modification enables a microcatheter to pass the lesion. E: exchanging to a 0.009-inch Rota-wire from a 0.014-inch conventional wire. F: high-speed rotational atherectomy is performed.

Our case demonstrates a successful combination therapy of ELA and high-speed RA in severely calcified SFA-CTO. The modification of the CTO entry by ELA complemented the disadvantage of the need for the dedicated wire in RA and aided in crossing the microcatheter to the distal CTO. In fact, this combined strategy is known as the RASER (LASER and high-speed Rotational atherectomy) technique in coronary section [15, 16]. After the wire successfully crosses the severely calcified lesion, a microcatheter often cannot pass the lesion. Subsequently, ELA ablates the only CTO entry. This modification enables the microcatheter to pass the lesion. RA can ablate the CTO body after retrieving a 0.014-inch conventional wire and crossing a 0.009-inch Rota-wire (Figure 6).

This is a treatment option for severely calcified lesions. To the best of our knowledge, the RASER technique in EVT section has not been reported in literature.

Although the above technique is technically feasible and beneficial, it should be considered as a last-resort technique in case of complications, like uncontrollable bleeding or absolute contraindications of surgical revascularization, because both ELA and RA in EVT are considered off-label use in Japan.

## 4. Conclusion

Combined therapy with ELA and high-speed RA in EVT section is effective in CTO of the SFA and could be a useful option in managing severely calcified lesions.

## Supplementary Materials

Supplementary Materials Video Clip S1: first angiogram. Digital angiography shows severe calcification of the right superficial femoral artery and the chronic total occlusion of the middle superficial femoral artery. Video Clip S2: rendezvous technique. The retrograde wire progresses into the antegrade child catheter. Video Clip S3: final angiogram of the proximal superficial femoral artery. Video Clip S4: final angiogram of the distal superficial femoral artery.
